# Short or Long Interval between Priming and Boosting: Does It Impact on the Vaccine Immunogenicity?

**DOI:** 10.3390/vaccines9030289

**Published:** 2021-03-20

**Authors:** Elena Pettini, Gabiria Pastore, Fabio Fiorino, Donata Medaglini, Annalisa Ciabattini

**Affiliations:** Laboratory of Molecular Microbiology and Biotechnology (LA.M.M.B.), Department of Medical Biotechnologies, University of Siena, 53100 Siena, Italy; pettini5@unisi.it (E.P.); gabiria.pastore@unisi.it (G.P.); fiorino4@unisi.it (F.F.); donata.medaglini@unisi.it (D.M.)

**Keywords:** immunization, alum, B cell response, T cell response, antibodies, prime–boost schedules

## Abstract

Characterizing the impact of the vaccination schedule on the induction of B and T cell immune responses is critical for improving vaccine immunogenicity. Here we compare the effect of a short (4 weeks) or a long (18 weeks) interval between priming and boosting in mice, using a model vaccine formulation based on the chimeric tuberculosis vaccine antigen H56 combined with alum. While no significant difference was observed in serum antigen-specific IgG response and the induction of antigen-specific T follicular helper cells into draining lymph nodes after the two immunization schedules, a longer interval between priming and boosting elicited a higher number of germinal center-B cells and H56-specific antibody-secreting cells and modulated the effector function of reactivated CD4+ T cells. These data show that the scheduling of the booster immunization could affect the immune response elicited by vaccination modulating and improving the immunogenicity of the vaccine.

## 1. Introduction

The efficacy of most preventive vaccines relies on both the antibody response to block pathogen infection and the generation of immune memory cells capable of rapid and effective reactivation following pathogen re-exposure. Most vaccines are administered in two or more doses, the first one necessary for priming the immune system and generating cells able to fight the infection, such as plasma cells releasing antibodies, and the second one that boosts the primary response increasing the quality and the magnitude of the pathogen-specific immune response. This approach gives rise to the concept of prime–boost that is important not only in terms of improving the magnitude and duration of the response but also the quality.

In the context of vaccination strategies, the antigen dose, the choice of the adjuvant or delivery system, the selection of homologous/heterologous vaccination regiments, the immunization route, and the time-intervals between priming and boosting deeply affect the vaccine’s immunogenicity [1,2,3,4,5,6,7,8,9]. The priming event is crucial for imprinting the host immune response. We have previously demonstrated that primary immunization with protein subunit antigens and adjuvant deeply impacts the immunological signature of the vaccine, programming the host immune response [2,10]. Specific adjuvants can be selected for priming a desired immune response and eventually be boosted with a different vaccine formulation to optimize the vaccine antigen-specific immune response [2].

While prime–boost strategies were traditionally based on the administration of the same vaccine formulation multiple times over a given period (homologous vaccine prime–boost ), the current state of the art suggests that different types of vaccines containing the same antigen but different delivery systems (heterologous vaccine prime–boost ) can be more immunogenic [11]. The heterologous prime–boost strategy can be particularly promising when the vaccination schedule includes vaccine viral vectors, and the secondary response to the vaccine vector itself could downmodulate the booster efficacy. This is the case with a recombinant viral-vectored vaccine that may be impeded by vector-specific immunity induced during the priming immunization [12]. When a recombinant virus is used, the vaccination strategy can be based on a single immunization, particularly when replicating viral vectors are used [13], or heterologous virus vector for the boosting dose [1,14,15,16], or boosting after a longer time interval [17]. Over the last decade, the need to make predictions and model biological systems has acquired increasing importance [18,19]. Computational models simulating the kinetics of the immune response in different immunization conditions or the dynamics of a disease spread in the population have also been developed and can be a useful tool to generate in silico predictions that can then be experimentally confirmed [20,21].

The optimal time interval between priming and boosting in a vaccination schedule could be affected by many factors, including intrinsic factors, such as genetics, sex, resident microbiome, and age of the target population, as well as vaccine-related factors [22,23,24]. The prime–boost interval in a vaccination schedule may, in turn, significantly influence both the short- and long-term immune response to the vaccination and is often very challenging to determine. This aspect has been studied in some pre-clinical [25,26] and clinical studies [27,28,29,30,31,32,33]. During the actual SARS-CoV-2 pandemic situation, the timing between priming and boosting has emerged as a critical aspect of the vaccination program. The non-replicating viral-vector-based coronavirus vaccine candidate developed by the Oxford-AstraZeneca company has been tested in clinical trials, including different spacing between doses, finding that a longer gap (two to three months) led to a greater immune response [34]. Efficacy was higher if the second dose came 8 to 11 weeks after the first, and it was further increased among volunteers who received the second dose of vaccine more than 11 weeks after the first. Data suggest that dosing interval was one of the most significant factor in determining the efficacy of this vaccine. While there is some evidence from trials of the Oxford/AstraZeneca vaccine, the benefits of delaying the second dose of the mRNA vaccines are less clear given the lack of data. Nevertheless, in the United Kingdom (UK), the coronavirus vaccination schedule has been changed in respect of the approved protocol. To prioritize the administration of the first dose to as many people as possible, the interval between the two doses has been prolonged to as much as 12 weeks, even though the interval tested in clinical trials for the Pfizer-BioNTech and Moderna COVID-19 vaccines was 21 and 28 days, respectively.

The timing between priming and booster doses is, therefore, a critical aspect for the induction of the immune response that needs to be deeply analyzed in all of its components, ranging from the evaluation of the immediate effector response, measured in terms of circulating antigen-specific antibodies, to the long-term characterization of the memory cells, necessary for protecting the host over time.To this aim, studies in animal models offer the advantage of characterizing the different immune components not only in the blood but also within specific lymphoid compartments, such as the lymph nodes draining the immunization site, and to follow the kinetics of the response at multiple time points.

Here we tested in mice the effect of a short (4 weeks) or a long (18 weeks) interval between priming and boosting, using a model vaccine formulation based on a soluble vaccine antigen, the chimeric tuberculosis vaccine antigen H56 [35,36] and alum, the most widely used vaccine adjuvant [37,38]. The effect of the two different intervals was measured in terms of systemic antibody production, induction, and persistence of germinal center B cells and antigen-specific T helper cells, particularly T follicular cells, within the draining lymph nodes, as well as antibody-secreting cells and cytokine production in the spleen. The analysis showed that the interval between priming and boosting affects mainly the B-cell response, but not the systemic antibody response and the recall of antigen-specific CD4^+^ T cells within the draining lymph nodes.

## 2. Materials and Methods

### 2.1. Mice

Seven-weeks old female C57BL/6 mice, purchased from Charles River (Lecco, Italy), were housed under specific pathogen-free conditions in the animal facility of the Laboratory of Molecular Microbiology and Biotechnology (LA.M.M.B.), Department of Medical Biotechnologies at University of Siena, and treated according to the national guidelines (Decreto Legislativo 26/2014). Experiments were planned and conducted utilizing the three R’s principles (Reduce, Replace, and Refine), which included environmental enrichment and nesting, veterinary oversight, numbers reflecting statistical significance, and the use of anesthesia followed by cervical dislocation for the sacrifice. All animal studies were approved by the Italian Ministry of Health with authorization n° 1004/2015-PR on 22 September 2015.

### 2.2. Immunizations

Mice were primed by the subcutaneous route (s.c.) at the base of the tail with vaccine formulations including the chimeric tuberculosis vaccine antigen H56 (2 µg/mouse; Statens Serum Institute, Copenhagen, Denmark), administered alone or combined with the aluminum hydroxide adjuvant (0.5 mg/mouse; hereafter alum, 2% alhydrogel, Brenntag Biosector, Frederikssund, Denmark). Animals were subcutaneously boosted with H56 (0.5 µg/mouse) at weeks 4 or 18. Vaccines were injected in a volume of 100 μL/mouse of sterile water for injectable preparations (Fresenius Kabi, Verona, Italy). Groups of mice were sacrificed at week 4 (W4), 7 (W7), 11 (W11), and 15 (W15) post priming and at day 10 (D10) and 49 (D49) post boosting.

### 2.3. Sample Collection and Cell Preparation

Blood samples were taken from individual mice by the temporal plexus or cardiac puncture at weeks 2, 4, 7, 11, 15, 18 after priming and at days 10, 28, and 49 post boosting for both vaccination schedules. Samples were incubated for 30 min at 37 °C, centrifuged at 1200× *g* at 4 °C for 10 min and sera were then collected and stored at −80 °C until analysis. Draining lymph nodes (sub iliac, medial, and external) and spleens were mashed onto 70 µm nylon screens (Sefar Italia, Torino, Italy) and washed two times in complete RPMI medium (cRPMI, Lonza, Verviers, Belgium) supplemented with 100 U/mL penicillin/streptomycin and 10% fetal bovine serum (Gibco, Grand Island, NY, USA). Samples were treated with red blood cell lysis buffer, according to the manufacturer’s instruction (eBioscience, San Diego, CA, USA).

### 2.4. Multiparametric Flow Cytometric Analysis

Cell samples from draining lymph nodes were incubated for 30 min at 4 °C in Fc-blocking solution (complete medium with 5 μg/mL of CD16/CD32 mAb [clone 93; eBioscience, USA]). Cells were stained with AF700-conjugated anti-CD45R/B220 (clone RA3-6B2; BD Biosciences), BV421-conjugated anti-GL-7 (clone GL-7; BD Biosciences, New York, NY, USA), PE-Cy7-conjugated anti-CD95 (clone Jo2; eBioscience), APC-conjugated anti-IgD (clone 11–26c.2a; BD Biosciences), APC-conjugated anti-IgM (clone II/41; BD Biosciences), PE-conjugated anti-CD138 (clone 281-2; BD Biosciences), BV605-conjugated anti-CD3 (clone 17A2; Biolegend, San Diego, CA, USA), BV786-conjugated anti-CD19 (clone 1D3; BD Biosciences), PerCP-Vio700-conjugated anti-CD38 (clone 90,4; Milteny, San Diego, CA, USA). To evaluate the T cell response cells from draining lymph nodes were stained for 1 h at room temperature with PE-conjugated I-A(b) M. tuberculosis Ag85B precursor 280–294 (FQDAYNAAGGHNAVF) tetramer (kindly provided by NIH MHC Tetramer Core Facility, Emory University, Atlanta, GA, USA) together with BV650-conjugated anti-CXCR5 (clone 2G8, BD Biosciences, USA). Cells were washed and stained with HV500-conjugated anti-CD4 (clone RM4-5; BD Biosciences), APC-conjugated anti-CD44 (clone IM-7; Biolegend), BV786-conjugated anti-CD273 (PD-1, clone TY25; BD Biosciences). Samples were labeled with a Live/Dead Fixable Near IR Dead Cell Stain Kit, according to the manufacturer’s instruction (Invitrogen, Eugene, OR, USA). Intracellular cytokine production was assessed on splenocytes cultured for 6 h in the presence of anti-CD28, anti-CD49d (both 2 µg/mL, eBioscience), and H56 protein (2 µg/mL). Unstimulated or phorbol 12-myristate 13-acetate (PMA) and ionomycin calcium salt (50 ng/mL and 1 µM, respectively, Sigma–Aldrich, St Louis, MO, USA) treated cells were used as negative and positive controls, respectively. Brefeldin A (5 µg/mL, Sigma–Aldrich) and monensin solution (2µM, eBioscience) were added to all samples for the last 4 h of incubation. Cells were washed twice in PBS and labeled with a Live/Dead Fixable Yellow Stain Kit, according to the manufacturer’s instruction (Invitrogen, USA). Fixation and permeabilization were performed using a BD Cytofix/Cytoperm kit, according to the manufacturer’s instruction (BD Biosciences) before Fc-blocking and stained with HV500-conjugated anti-CD4 (clone RM4-5; BD Biosciences), BV786-conjugated anti-CD44 (clone IM-7; BD Biosciences), PerCP Cy5.5-conjugated anti-IFN-γ (clone XMG1.2; BD Biosciences), AF700-conjugated anti-TNF-α (clone MP6-XT22; BD Biosciences), APC-conjugated anti-IL-17A (clone eBio17B7; eBioscience), AF488-conjugated anti-IL-4 (clone 11B11; eBioscience), AF488-conjugated anti-IL-13 (clone eBio13A; eBioscience) and PE-conjugate anti-IL2 (clone JES6-5H4; BD Biosciences). All antibodies and tetramers were titrated for optimal dilution. About 5–10 × 10^5^ cells were stored for each sample and acquired on BD LSRFortessa X20 flow cytometer (BD Biosciences). Data analysis was performed using FlowJo v10 (TreeStar, Ashland, OR, USA).

### 2.5. B-Cell ELISPOT

Antibody secreting cells (ASCs) within splenocytes were evaluated by the mouse IgG Single-Color ELISPOT assay (CTL Europe GmbH, Bonn, Germany) 10 days post boost. Multiscreen filter 96-well plates were coated with H56 (5 μg/mL) for the detection of antigen-specific IgG or with anti-IgG capture antibody for the detection of total IgG overnight at 4 °C. Plates were washed and blocked for 30 min with a serum-free CTL-Test B culture medium (CTL, Europe GmbH) supplemented with 1% L-glutamine (Sigma–Aldrich, St. Louis, MO, USA). One million of cells/well were added in a volume of 100 μL of CTL-Test B medium for the analysis of H56-specific IgG ASCs in triplicate. Plates were incubated for 20 h at 37 °C with 5% CO_2_, washed with PBS-0.05% Tween 20, and added with anti-murine IgG Detection Solutions for 3 hours at room temperature. Plates were washed, incubated with 80 µL/well of Tertiary Solution Strep-AP (diluted 1:1000) at room temperature for 1 h, washed again, and added with Blue Developer Solution for 15 min. The number of spots was determined by plate scanning, and analysis was performed with an Immunospot S6 ULTIMATE Analyzer (CTL, Europe GmbH).

### 2.6. ELISA

Serum H56-specific IgG was determined by enzyme-linked immunosorbent assay (ELISA) at different time points. Flat bottomed Maxisorp microtiter plates (Nunc, Roskilde, Denmark) were coated with H56 (0.5 µg/mL) for 3 h at 37 °C or overnight at 4 °C in a volume of 100 µL/well. Plates were washed and blocked with 200 µL/well of Phosphate Buffered Saline (PBS) containing 1% Bovine Serum Albumin (BSA,Sigma–Aldrich) for 2 h at 37 °C. Serum samples were added and titrated in a two-fold dilution in duplicate in PBS supplemented with 0.05% Tween 20 and 0.1% BSA (diluent buffer) in 100 µL/well. After incubation for 2 h at 37 °C, samples were incubated with the alkaline phosphatase-conjugate goat anti-mouse IgG (diluted 1:1000 in a diluent buffer; Southern Biotechnology, Birmingham, AL, USA) for 2 h at 37 °C in 100 µL/well and developed by adding 1 mg/mL of alkaline phosphatase substrate (Sigma–Aldrich) in 200 µL/well. The optical density was recorded using Multiskan FC Microplate Photometer (Thermo Scientific, Waltham, MA, USA). Antibody titers were expressed as the reciprocal of the dilution of the sample, reporting the double optical density (OD) value compared to the background.

### 2.7. Statistical Analysis

All samples were tested individually. The number of serum antibodies was expressed as geometric mean titers (GMT) ± 95% CI, and statistical analysis of antibody titers was performed on log-transformed data. The Mann–Whitney test for multiple pairwise comparisons was used to assess the statistical difference between the immune response induced after the short or long vaccination schedules. A *p*-value ≤ 0.05 was considered significant. Statistical analysis was performed using Graph Pad Prism version 9 (GraphPad Software, San Diego, CA, USA).

## 3. Results

**Figure 1:**
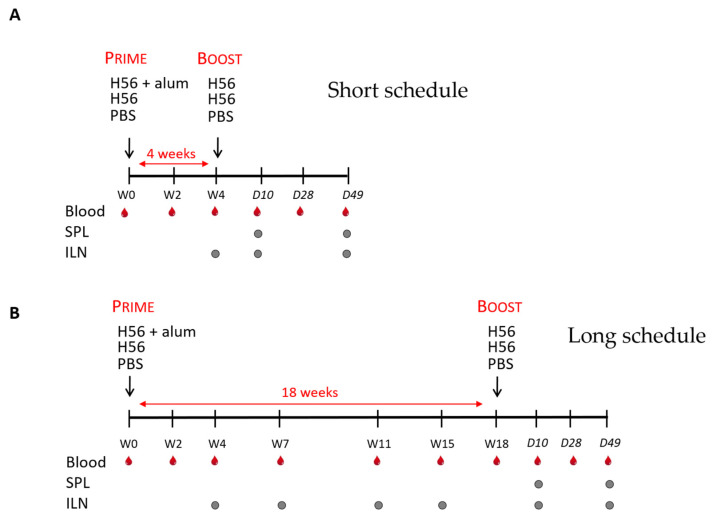
Experimental design. Two different immunization schedules (**A**,**B**) were tested and compared for the induction of serum antibodies (in blood), germinal center B cells, and antigen-specific T helper cells (in draining iliac lymph nodes, ILN), and cytokine production (in the spleen, SPL). C57BL/6 mice were subcutaneously immunized with the H56 antigen and alum and boosted with the antigen 4 (**A**) or 18 (**B**) weeks apart. Mice immunized with H56 antigen alone, or Phosphate Buffered Saline (PBS) were used as controls. Blood samples, ILN, and SPL were collected at the time points indicated by symbols. W, weeks; D, days.

In the present work, we tested the effect of a short (4 weeks) or a long (18 weeks) interval between priming and boosting, using a model vaccine formulation based on H56 vaccine antigen and alum, on antigen-specific T and B immune responses. Mice were subcutaneously primed with the H56 vaccine antigen combined with alum and boosted with a lower dose of antigen alone. Control groups were primed and boosted with the antigen alone or left untreated. The induction and persistence of antigen-specific antibodies, germinal center B cells, antigen-specific T helper cells, especially T follicular cells, as well as antigen-specific antibody-secreting B-cell and cytokine production were assessed and compared between the two schedules. A schematic representation of the experimental design is reported in Figure 1.

### 3.1. Humoral Immune Response

**Figure 2:**
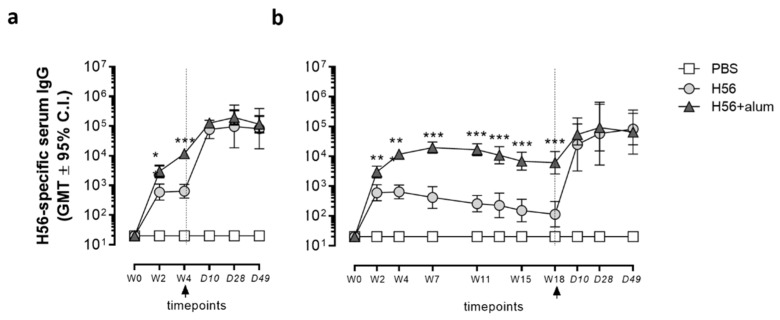
Antigen-specific IgG response. C57BL/6 mice were subcutaneously immunized, as summarized in Figure 1, and humoral response was analysed after the short (**a**) and long (**b**) immunization protocol. H56-specific IgG serum titers were analyzed 0, 2, 4, 7, 11, 15, 18 weeks following priming, and 10, 28, and 49 days after boosting by ELISA. Values are reported as GMT ± 95% CI of 12 mice from 2 different experiments. Antibody titers were expressed as the reciprocal of the dilution of the sample, reporting an optical density value double with respect to the background. The Mann–Whitney test for multiple pairwise comparisons was used for assessing statistical differences for each time point between groups. ** *p* ≤ 0.001; *** *p* ≤ 0.0001.

Antigen-specific IgG antibody response was evaluated at different time points after immunization with short (4 weeks, W4) or long (18 weeks, W18) intervals between priming and boosting (Figure 2). Serum H56-specific IgG levels rapidly increased 2 weeks after priming (GMT = 2500) and further augmented at week 4 (GMT = 11,000; Figure 2). In the longer schedule, H56-specific IgG peaked with a GMT = 19,600 seven weeks after priming (Figure 2b) and was stably maintained until week 18.

On the contrary, IgG elicited by priming with H56 antigen alone peaked at week 4 (GMT = 640) and then slowly declined to reach GMT = 115 at week 18 (Figure 2b). At all the time points before boosting, mice primed with H56 + alum showed a significantly higher IgG response compared to mice primed with H56 alone (*p* ≤ 0.01 at W2 and *p* ≤ 0.001 from W4 to W18; Figure 2). Boosting with H56 antigen at week 4 (Figure 2a) or 18 (Figure 2b) induced a significant rapid increase in IgG levels with respect to the pre-boost (*p* ≤ 0.001). Humoral H56-specific immune response was not significantly affected by the intervals between priming and boosting, resulting in similar levels of antigen-specific IgG 10 days after booster immunization (GMT = 126,000 and GMT = 54,000 in short and long schedules, respectively, with *p* = 0.06). No H56-specific humoral response was detected in mice that received PBS. Overall, the short and long intervals between priming and boosting had little effect on the serum antigen-specific IgG response after vaccination with the tested H56 + alum formulation.

### 3.2. B-Cell Response

**Figure 3:**
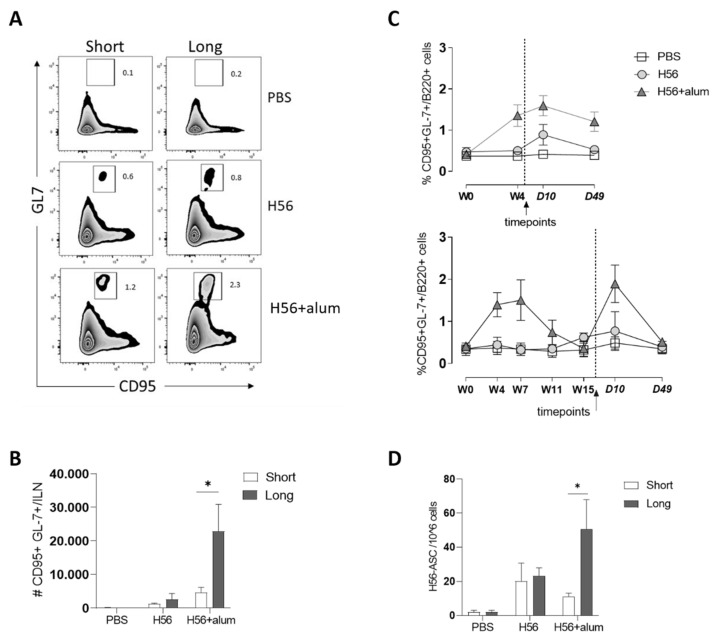
B-cell response. C57BL/6 mice were subcutaneously immunized, as summarized in Figure 1. (**A**) Germinal center B cells were identified as GL-7 + CD95+ among B220 + B cells in iliac lymph nodes 10 days after boosting. Dot plots are shown from a single animal representative of the group, and numbers indicate frequencies of CD95 + GL-7 + respect to B220 + cells B–C. Time-course analysis of the frequencies of GC- cells, with respect to B220+ B cells (**B**) and absolute numbers of GC-B cells per ILN (**C**) reported as mean ± SEM of six mice per group. (**D**) Number of H56-specific IgG-secreting cells per million splenocytes, reported as mean ± SEM of six mice per group. The Mann–Whitney test for multiple pairwise comparisons was used to assess the statistical difference between the short (4W) and long (18W) schedules for each vaccine formulation. * *p* ≤ 0.05.

The induction of the germinal center (GC)-reaction is considered an important biomarker of B memory response [39] since within the GC, high-affinity B cells are selectively expanded and induced to differentiate into memory B cells and antibody-producing plasma cells [40]. Therefore, the induction and the persistence of GC-B cells were analyzed within the lymph nodes draining the immunization site after the short and long immunization schedules (Figure 3).

Germinal center B cells were identified by the co-expression of GL-7 and CD95 on B220+ cells. Representative dot plots showing the frequencies of GL-7 + CD95 + B cells are shown in Figure 3A. The comparison of the GC-B cells induced by the two vaccine schedules showed a significantly higher number of GL-7 + CD95+ B cells in mice primed with H56 + alum and boosted after a longer interval (W18) with respect to the shorter schedule (23,000 and 4500 cells per iliac lymph node, respectively; *p* ≤ 0.05; Figure 3B). The same trend was observed in mice primed with the H56 vaccine antigen alone, with 2500 and 1300 GC-B cells per lymph node in the long and short schedules, respectively (Figure 3B). No GL-7 + CD95 + B cells were observed in mice that received PBS as control (Figure 3A,B).

The kinetic analysis of the GC reaction after primary immunization showed that when the booster immunization was performed 4 weeks after priming, the response was still in the effector phase with high frequencies of GL-7 + CD95+ B cells. However, in the longer time interval schedule, the reaction declined before the booster immunization (Figure 3C). At all time points analyzed, the presence of the alum adjuvant in the vaccine formulation used for priming induced a higher number of GC-B cells with respect to H56 alone in both the vaccine schedules assessed (Figure 3). No GC-B cells were detected in PBS control mice.

To further characterize the B-cell response, the number of H56-specific antibody-secreting cells (ASCs) was analyzed in the spleen at D10 post boost after the two vaccination schedules. A significantly higher number of H56-specific IgG secreting cells was detected in mice primed with H56 + alum after the longer schedule with respect to the shorter one (50 and 10 ASCs/10^6^ splenocytes, respectively; *p* ≤ 0.05; Figure 3D). No differences were detected between the W4 and W18 schedules concerning the number of ASCs detected in mice primed with the antigen alone or in PBS-treated mice (Figure 3D). Taken together, these results suggest that a longer interval between priming and boosting potentiates the B-cell response, eliciting a higher number of GC-B cells and ASCs.

### 3.3. Ag-Specific CD4+ T Cell Response

**Figure 4:**
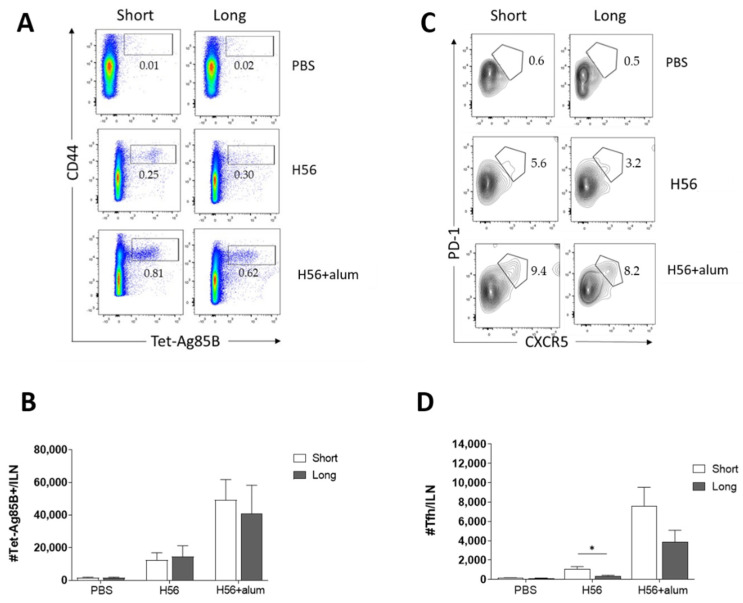
Induction of Ag85B-specific CD4+ T cells. C57BL/6 mice were subcutaneously immunized, as summarized in Figure 1, and lymph nodes draining the sites of immunization (ILN) were collected 10 days after boosting. Ag-specific T cells were identified by staining with Ag85B-specific MHC class II tetramers (Tet-Ag85B). Tetramer+ T cells, detected as CD44high Tet-Ag85B+ cells, gated on live CD4+ lymphocytes (**A**), and the follicular helper T cells (Tfh), identified as CXCR5+ PD-1+ among tetramer-binding CD4+ T cells (**B**), are shown from a single animal representative of the group. Absolute numbers of Tet-Ag85B+ CD44+ T cells (**C**) and follicular helper T cells (**D**) per ILN elicited by the two schedules assessed, reported as mean ± SEM of six mice per group. The Mann–Whitney test for multiple pairwise comparisons was used to assess the statistical difference between the short (4W) and long (18W) schedules for each vaccine formulation. * *p* ≤ 0.05.

The induction of antigen-specific CD4+ T cells into the iliac draining lymph nodes was analyzed 10 days after the boosting with the two immunization schedules. CD4+ T cells specific for the immunodominant epitope of Ag85B, which is part of the chimeric H56 protein, were identified using Ag85B280-294-complexed MHC class II tetramers. Staining specificity was determined using a control tetramer complexed with an unrelated antigen that showed a level of staining below 0.02% (data not shown). Representative dot plots showing the frequencies of tetramer-positive (Tet-Ag85B+) T cells elicited by different vaccine schedules are shown in Figure 4A.

A similar number of helper Tet-Ag85B+ T cells was induced with both 4- and 18-weeks interval between priming and boosting, in mice primed with H56 + alum (Figure 4A,B). The presence of the adjuvant in the priming formulation induced a higher number of Tet-Ag85B+ CD4+ T cells with respect to antigen alone. The presence of T follicular helper cells (Tfh), a specialized CD4+ T subset that participates in GC development and contributes to the processes of affinity maturation and class switching of antibodies [41,42], was investigated within Tet-Ag85B+ T cells (Figure 4C,D). Tetramer-positive CD4+ T cells with a phenotype of Tfh cells (PD-1high CXCR5high) were identified in draining lymph nodes 10 days after booster immunization (Figure 4C,D). A higher number of H56-specific Tfh was detected in mice primed with the H56 + alum and boosted 4 weeks later respect to mice boosted 18 weeks later (7625 and 3900 Tfh cells per lymph node, respectively), and a similar trend was observed in mice primed and boosted with the antigen alone (1000 and 350 Tfh cells per lymph node, respectively; *p* ≤ 0.05; Figure 4D).

These results show that the induction of antigen-specific T helper cells, including the T follicular helper ones, is not deeply affected by the weeks of the interval between priming and boosting.

### 3.4. CD4+ T Cell Multifunctional Profile

**Figure 5:**
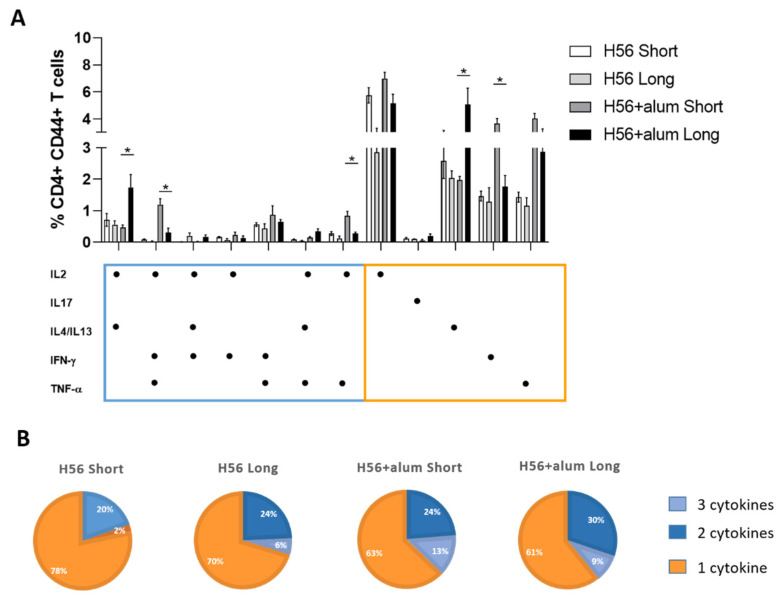
Helper T cell multifunctional response. Multifunctional profiles of CD4+ T cells detected by the different immunization protocols as reported in Figure 1. (**A**) Histograms represent the frequency of CD4+ CD44+ T cells producing different combinations of cytokines after the short and long schedule. Responses are grouped and color-coded according to the functionality (orange for single cytokine, light blue for two or three cytokine production). Values are reported as mean ± SEM of six mice per group. The Kruskal–Wallis test followed by Dunn’s post hoc test for multiple comparison was used to assess statistical differences among groups (*p ≤ 0.05). (**B**) Pie charts represent the portion of CD4+ T cells producing three cytokines (orange), two cytokines (light blue), or a single one (grey). Frequencies are reported within each slice.

To have a picture of the multifunctional profile of T cells elicited by immunization with the short or long intervals between priming and boosting, a Boolean analysis of intracellular cytokine production was performed for assessing the frequency of cells producing one (single), two or three (multifunctional) cytokines. As shown in Figure 5, the frequency of activated CD4+ T cells producing IL-4+/IL-13+ alone or with IL-2, indicative of a Th2 response, was significantly higher in mice primed with the H56 + alum and boosted with the long schedule with respect to the one boosted with the short schedule (5% and 1.7% compared to 2% and 0.5%, respectively *p* ≤ 0.05; Figure 5A).

On the contrary, when mice were boosted with the short schedule a significantly higher production of multifunctional IL-2^+^IFN-γ^+^TNF-α^+^ (1.15%), IL-2^+^TNF-α^+^ (0.8%) as well as single IL-2^+^ (7%) and IFN-γ^+^ (3.6%) cells was observed compared to the long schedule (0.1%, 0.8%, 5%, and 1.7% respectively; *p* ≤ 0.05; Figure 5A). The presence of alum in the vaccine formulation used for priming generally induced higher frequencies of multifunctional CD4^+^ CD44^+^ T cells, as reported in Figure 5B, with respect to antigen alone, and the antigen alone needed the long schedule to enhance the polyfunctional helper T cells.

In summary, a significant Th2 response was matured only after a longer interval between priming and boosting, while a higher frequency of polyfunctional or IFN-γ^+^ Th1 cells was elicited by a shorter interval.

## 4. Discussion

In this study, we sought to investigate how the timing of the booster dose administration impacts the induction of B and T cell immune responses. Here we showed that: (i) the longer (18 weeks) schedule enhanced the B-cell response stimulating a higher number of GC-B cells and H56-specific ASCs; (ii) no significant differences were found in serum antigen-specific IgG titers after the short (4 weeks) or long (18 weeks) intervals between priming boosting; (iii) a similar number of antigen-specific CD4+ T cells was recalled by both short or long schedules, but their effector function was different.

Most vaccines are administered in two or more doses [43], with the first one deeply affecting the magnitude and the quality of the B and T-cell responses, and the booster dose, administered sometime later, which increases the immune response and stimulates the production of higher affinity antigen-specific antibodies. The use of multiple vaccine doses has proven to be essential in providing high levels of protection against many diseases preventable with vaccination. However, the effectiveness of vaccination depends on several key factors, among which is the optimal scheduling of a booster dose since it could significantly affect the long-term disease dynamics. Shortening the vaccine schedule in the face of a new emerging infectious disease or outbreak, as for the current SARS-CoV-2 pandemic, is certainly an attractive possibility to ensure faster protection of the population at risk, but an accelerated vaccination schedule could negatively impact the humoral immunity [21].

The impact of the interval between priming and boosting on the intensity and quality of the specific antibody response has also been studied in clinical studies showing that a gap shorter than 3 weeks between immunization impaired the ability to develop protective immunity against smallpox after Modified vaccinia Ankara immunizations [29]. In addition, in the RV 114 HIV vaccine trial, using the viral vector ALVAC-HIV that contains genetically engineered versions of three HIV genes (env, gag, and pol), it was demonstrated that longer intervals between priming and boosting improved humoral response [31]. Despite the importance of dosing interval between primary and booster vaccination, the impact of such interval on the induced T and B cell immune response still remains to be elucidated.

In our study, mice were primed with a model vaccine formulation constituted by a soluble vaccine antigen combined with alum, the adjuvant with the longest and most widespread clinical usage and boosted with a lower dose of vaccine antigen to select antigen-specific clones of T and B cells at two different time intervals. Significant differences in circulating antibody titers elicited by the two schedules were not observed. This suggests that the stimulation of effector B cells was not significantly affected by the timing of booster administration, probably as a consequence of the strong immunogenicity of this vaccine formulation, which elicited an antibody response with significantly high IgG titers persisting for 18 weeks after a single immunization. As already observed in previous studies, primary immunization with H56 and alum elicits IgD^−^B220^int^CD138^+^ plasma cells in draining lymph nodes 12 days after priming [2]. These cells, rapidly generated after antigenic stimulation, are generally defined as short-term plasma cells and develop in the extrafollicular reaction [40]. Nevertheless, the persistence of the humoral primary response detected in the longer booster schedule demonstrates that active plasma cells are maintained for many weeks in the absence of a booster immunization. Since during the course of the primary response, the germinal center reaction also clearly develops, with a peak of GC-B cells after 7 weeks as demonstrated here, we can speculate that plasma cells, possibly generated inside the germinal center reaction and subsequently disseminated to the bone marrow as long-lived plasma cells, can contribute to the maintenance of the high levels of serum antibody response.

When compared to the immune response elicited by the H56 antigen alone, this analysis also confirms the key role of the adjuvant in strongly reprogramming the immune response [10] and potentiating the vaccine immunogenicity as we also previously observed with other vaccine adjuvants [3,44], while no significant differences were observed in the antigen-specific IgG titers at the time points analyzed after the booster immunization.

From a vaccination perspective, it is crucial to stimulate immunologic memory, generating antigen-specific memory B cells capable of responding to the vaccine antigen more quickly and robustly, and plasma cells secreting antigen-specific antibodies. The germinal center reaction yields high-affinity antibody-secreting plasma cells and memory B cells, both critical for host protection during secondary challenge with pathogens [39]. The duration of the germinal center reaction may vary depending on the nature of the antigen and may last for several weeks or months, and GC cells persist for three to four weeks, as we have also observed in our study. Thereafter, memory B cells proliferate in follicles in a T-cell mediated manner for another few weeks. The timing of the secondary immunization could impair the differentiation pathways of memory B and T cells [33]. In general, to generate memory T cells with high proliferative potential and memory B cells that have to go through the germinal center reaction and take several months to develop, it is better to have an interval of at least 2–3 months between the prime and the boost [42]. This was recently discussed with an HIV envelope trimer-based candidate vaccine evaluated in nonhuman primates. The study was demonstrated that longer intervals between prime and booster immunizations increased GC B cell frequencies [25,26]. Recent studies have also suggested that increasing the time intervals between immunizations during a prime–boost protocol may permit increased differentiation of effector memory cells to central memory cells before boosting and thereby enhance the effectiveness of the immunizations [17]. The short (4 weeks) schedule assessed in this study likely prevented the GC reaction from developing fully, and in addition, the booster dose was not administered during the late stages of the effector to memory transition, as happened with the longer schedule. Further studies aimed at investigating how memory cells elicited by the shorter or longer immunization schedules will react to the recall antigen restimulation will be necessary.

In the context of vaccination strategies, the induction of effector T cell immune responses is also critical for improving the vaccine immunogenicity as well as for modulating the quality of the immune response [1]. Here, it was observed that when the booster immunization was delayed to 18 weeks, reactivated CD4+ T were differentiated into Th2 cells, producing IL-4^+^/IL-13^+^ cytokines. Differently, higher frequencies of pro-inflammatory polyfunctional (IFN- γ^+^ TNF-α^+^ IL-2^+^) Th1 cells were elicited when mice were boosted earlier at 4 weeks after priming. This could suggest that the impact of the booster immunization on the T cell response may depend on the status of the T cells themselves. Indeed, after antigen encounter, T cells undergo clonal expansion and differentiation, followed by a contraction phase once the antigenic stimulation has been cleared [45]. During these phases, T cell response transits from an effector to a memory phase [46]. It is, therefore, possible that the effect of the booster immunization can vary according to the effector status of the antigen-specific T cells. At the same time, the number of recalled antigen-specific CD4+ T cells or follicular T cells within the draining lymph nodes was not affected by the timing of the booster dose.

Taken together, these data provide a picture of the impact of the timing of the booster administration on the B and T cell responses elicited by a vaccine formulation based on soluble antigen and alum, with a complete analysis of the kinetic of the primary response. In this context, the timing of the booster immunization was shown to affect the B-cell response, with an increasing number of GC B cells and antigen-specific antibody-secreting B cells when administered at a longer interval (18 weeks) compared to the shorter one (4 weeks), and the effector function of the recalled CD4+ T cell response. On the contrary, the delay of the booster administration did not profoundly affect the systemic antibody responses and the number of recalled antigen-specific CD4+ and follicular T cells within the draining lymph nodes. These results reflect the complexity and the heterogeneity of the immune response towards a specific vaccine formulation and highlight that the interval between priming and boosting can differently impact some effector mechanisms, which can vary according to the vaccine platforms used.

These data offer valuable indications that the timing of the booster dose plays a critical role in shaping vaccine immunogenicity, improving the efficacy of prime–boost vaccination strategies.
